# Beyond Body Mass Index: A Systematic Review and Meta-Analysis of Adiposity Measures Versus Body Mass Index in Predicting Infection Risk Following Elective Spinal Surgery

**DOI:** 10.7759/cureus.89970

**Published:** 2025-08-13

**Authors:** Khong Wee Lee, Khairina Khairuddin, Ashwani Nugur, Naveen Vijayasingam

**Affiliations:** 1 Orthopaedics, Ysbyty Gwynedd, Bangor, GBR; 2 Public Health, Birmingham City University, Birmingham, GBR; 3 Trauma and Orthopaedics, Ysbyty Gwynedd, Bangor, GBR; 4 Trauma and Orthopaedics, Tawau General Hospital, Sabah, MYS

**Keywords:** adiposity metrics, body mass index, postoperative infection, preoperative assessment, risk stratification, spinal fusion surgery, subcutaneous fat thickness, surgical site infection

## Abstract

Postoperative infections are among the most frequent complications following elective spinal surgery, often leading to reoperations, prolonged hospitalisation, and increased healthcare burden. Body mass index (BMI), although widely used to assess obesity-related surgical risk, lacks anatomical specificity and fails to capture regional fat distribution relevant to wound healing and operative exposure.

This systematic review adhered to Preferred Reporting Items for Systematic Reviews and Meta-Analyses (PRISMA) guidelines and evaluated observational studies comparing BMI with site-specific adiposity metrics in predicting surgical site infections (SSI) following elective spinal fusion procedures. Searches were conducted across five databases using predefined terms related to spinal surgery, adiposity, BMI, and infection outcomes. Inclusion criteria targeted studies reporting postoperative SSI alongside a comparative analysis of BMI and regional fat measures. Data extracted included sample size, adiposity definitions, infection outcomes, and statistical effect sizes. A subgroup meta-analysis was performed using a random-effects model to pool odds ratios (ORs) from studies evaluating subcutaneous fat thickness.

Seven studies met the eligibility criteria. Across all studies, localised adiposity measures showed stronger associations with postoperative SSI than BMI. In a subgroup meta-analysis of three studies reporting subcutaneous fat thickness, the pooled OR was 1.09 (95% CI: 1.01-1.17; p=0.0193), indicating a statistically significant increased odds of infection among patients with greater regional fat burden. Significant statistical heterogeneity was observed (I²=84%).

Site-specific adiposity metrics, particularly subcutaneous fat thickness, are more reliable predictors of postoperative infection than BMI in spinal surgery populations. Their integration into preoperative risk assessment tools could enhance surgical planning and infection prevention. Future research should focus on standardising adiposity measurement protocols and validating threshold values across diverse spinal procedures.

## Introduction and background

Postoperative infection remains a significant challenge in elective spinal surgeries, contributing to increased patient morbidity, prolonged hospitalisation, and heightened resource utilisation [1]. Accurate preoperative risk assessment is therefore pivotal to surgical decision-making and preventative strategies [2]. Among the most widely adopted metrics for obesity and surgical risk is body mass index (BMI), primarily due to its accessibility and entrenched presence in clinical practice [3]. However, its anatomical limitations and poor discriminatory power have led researchers to question its utility in predicting postoperative complications, particularly in surgeries where regional fat distribution may directly impact operative exposure and wound healing.

Rothman [4] emphasised that BMI, as a proxy for adiposity, suffers from substantial measurement error, poor sensitivity, and a tendency toward misclassification. It fails to differentiate lean mass from fat mass, obscures the contribution of visceral and subcutaneous fat, and overlooks the anatomical relevance of fat distribution over the surgical field. In the context of spinal surgery, where the depth and composition of overlying tissues influence both surgical access and tissue recovery, this lack of specificity can undermine preoperative planning and risk stratification.

These limitations become even more pronounced in functionally complex populations. As highlighted by Sukkarieh et al. [5], BMI may be particularly unreliable in non-ambulatory paediatric patients with neuromuscular scoliosis. Accurate height measurements, a critical component of BMI calculation, are often unattainable due to severe spinal deformities and limb contractures. Sukkarieh et al. [5] instead proposed the use of radiographically derived subcutaneous fat thickness ratios, demonstrating their correlation with surgical site infection (SSI) risk in posterior spinal fusion procedures. Their study not only challenged BMI's validity in this population but also illustrated how anatomically localised adiposity metrics can offer a more clinically meaningful alternative.

This systematic review investigates whether such alternative adiposity measures, including subcutaneous fat thickness, skinfold ratios, and fat depth estimations, offer superior predictive power over BMI in forecasting postoperative infection across elective spinal surgeries. By critically examining existing literature and identifying methodological patterns, the review aims to inform future risk modelling practices and explore more anatomically precise tools for surgical stratification.

## Review

Methods

Following registration with the International Prospective Register of Systematic Reviews (PROSPERO ID: CRD420251121926), this systematic review was conducted following the Preferred Reporting Items for Systematic Reviews and Meta-Analyses (PRISMA) guidelines [6]. A comprehensive search was carried out across five electronic databases: PubMed, Embase, PsycINFO, Cochrane Library, and Scopus. Boolean combinations of keywords were used to identify relevant studies addressing spinal surgery, adiposity metrics, BMI, and postoperative infection outcomes. To ensure completeness, grey literature, conference abstracts, and citation lists of eligible studies were manually screened.

The search strategy was framed around a PICO approach, combining Population (P), Intervention (I), Comparator (C), and Outcome (O) keywords. The full list of Boolean terms is summarised in Table 1.

**Table 1:** The search steps

Search number	Search terms
Search 1 (P)	"spinal surgery" OR "spinal fusion" OR "lumbar fusion" OR "cervical fusion" OR "elective spine procedure"
Search 2 (I)	"subcutaneous fat thickness" OR "adiposity ratio" OR "local adiposity" OR "site-specific adiposity"
Search 3 (C)	"Body Mass Index" OR "BMI"
Search 4 (O)	"surgical site infection" OR "SSI" OR "postoperative infection" OR "wound complications"
Search 5	Search 1 AND Search 2 AND Search 3 AND Search 4

Eligible studies included adult patients undergoing elective cervical or lumbar spinal fusion procedures, with postoperative SSI outcomes defined according to the Centers for Disease Control and Prevention (CDC) criteria [7]. Each study was required to provide a direct statistical comparison between BMI and at least one local adiposity measure and to report sufficient statistical data for extraction, such as odds ratios (ORs), confidence intervals, p-values, and sample size. Only peer-reviewed articles published in English were considered. Exclusion criteria included studies of paediatric patients, trauma or emergency spinal surgeries, reviews and editorials, duplicate publications, lack of SSI data, or absence of adiposity metrics beyond BMI.

**Figure 1:**
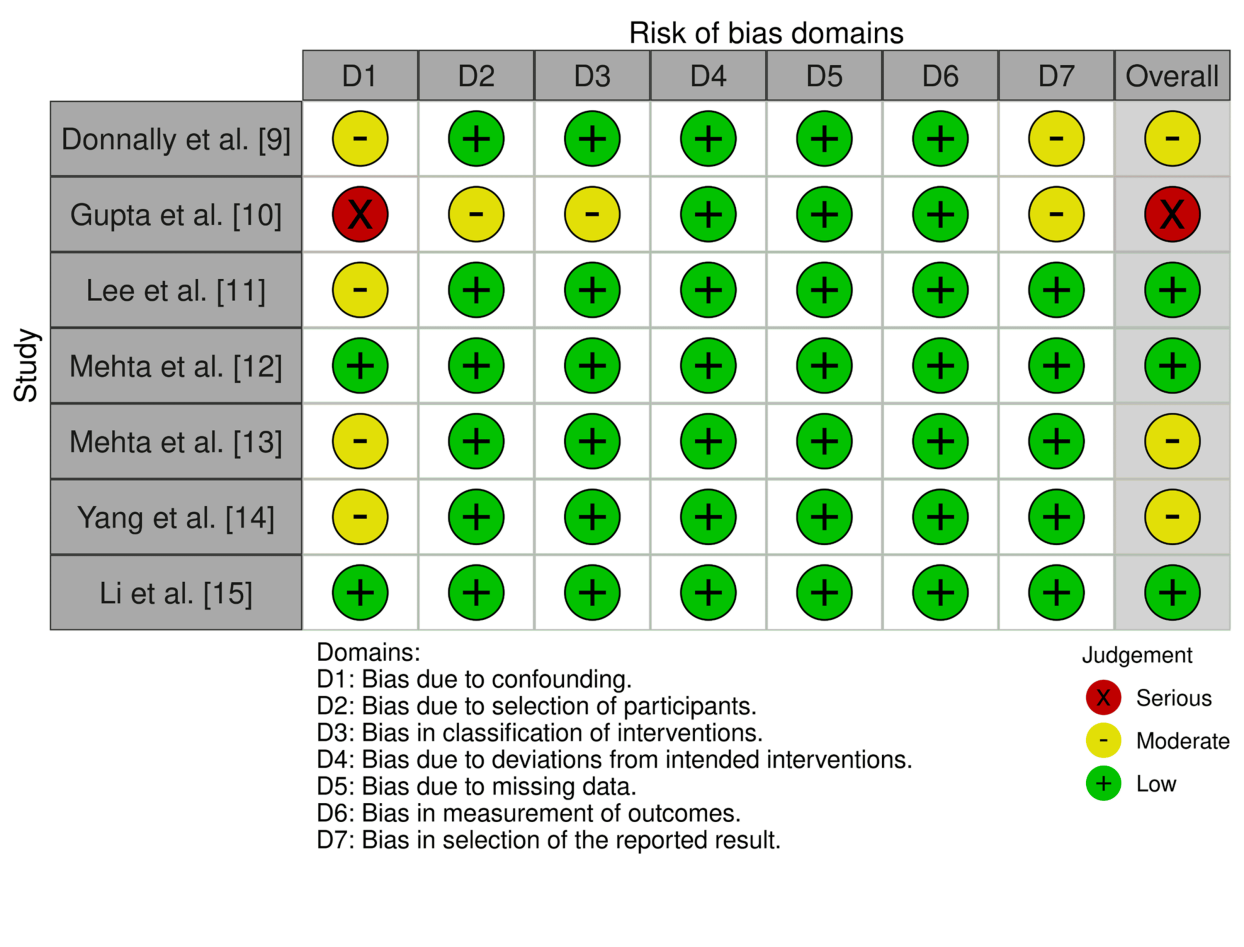
Cochrane ROBINS-1 risk of bias assessment across the included studies ROBINS-1: Risk of Bias in Non-randomised Studies

Two reviewers independently extracted study-level data, including demographic characteristics, adiposity definitions, surgical approach, statistical methodology, and infection outcomes. Disagreements were resolved through consensus. The Cochrane Risk of Bias in Non-randomised Studies (ROBINS-1) tool [8] was used to assess the quality of all eligible studies [9-15], as demonstrated in Figure 1.

A subgroup meta-analysis was conducted on three studies, namely, Lee et al. [11], Mehta et al. [12], and Li et al. [15], which investigated subcutaneous fat thickness in relation to SSI following lumbar spine procedures. Due to variability in measurement techniques and adiposity thresholds, a DerSimonian-Laird random-effects model [16] was applied. ORs were log-transformed, and between-study heterogeneity was quantified using the I² statistic to produce a pooled effect estimate [17].

Results

**Figure 2:**
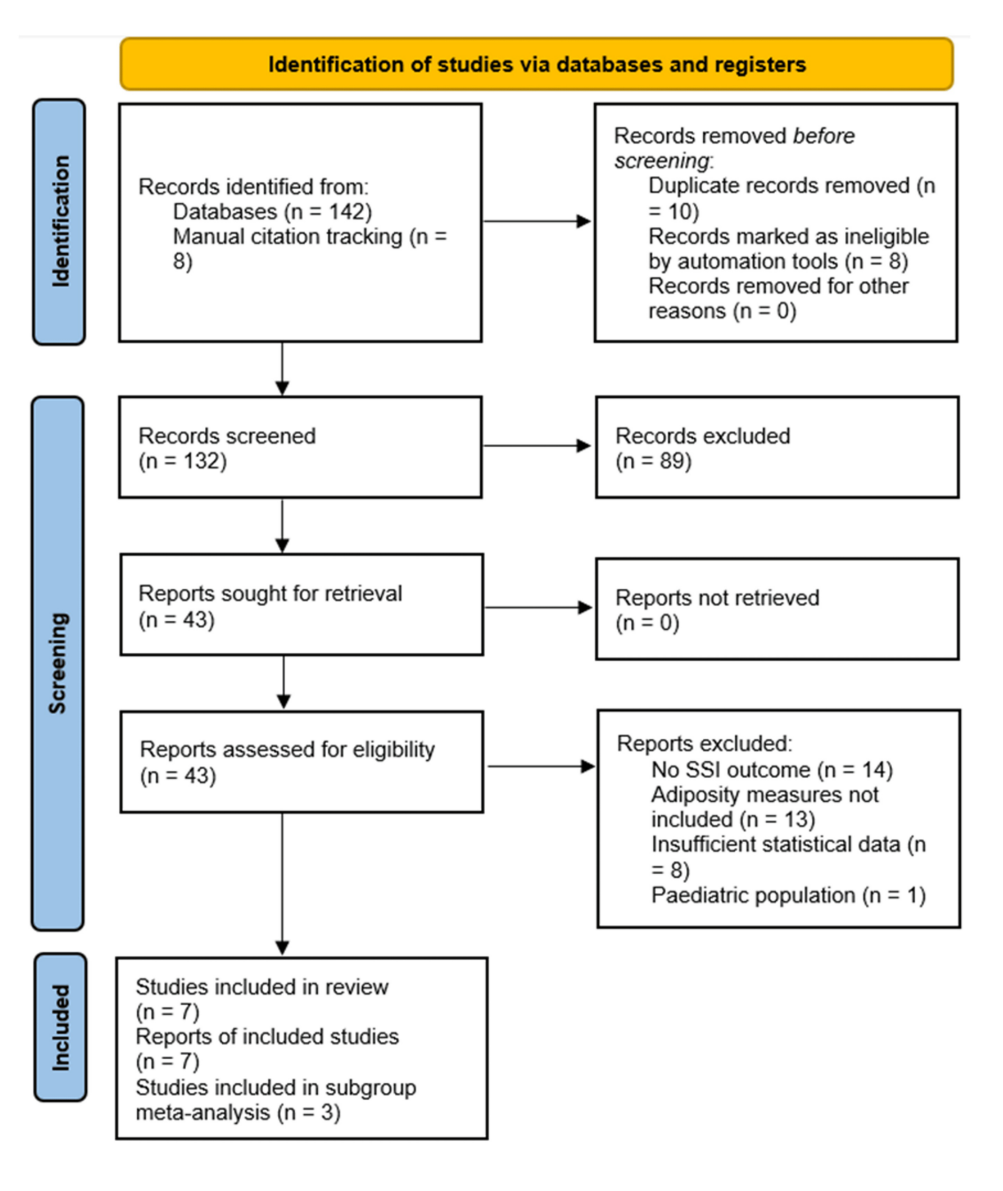
PRISMA sheet for final studies identified for review PRISMA: Preferred Reporting Items for Systematic Reviews and Meta-Analyses; SSI: surgical site infections

The study selection process is illustrated in Figure 2, which presents the PRISMA flow diagram for identifying final studies included in this review. After applying inclusion and exclusion criteria, seven articles were selected for qualitative synthesis, and one was excluded [5] due to its exclusive focus on paediatric patients with neuromuscular scoliosis.

To compare how BMI and localised adiposity metrics correlate with SSI outcomes, key findings from the included studies are summarised in Table 2. This overview covers surgery types, BMI and adiposity-related results, infection definitions, and major conclusions. Across cervical and lumbar spinal procedures, site-specific fat measurements such as subcutaneous thickness, lamina-skin distance, and adiposity ratios consistently demonstrated stronger predictive value for SSI than BMI. BMI was either non-significant or failed to retain significance in multivariate models.

**Table 2:** Study comparison of BMI versus adiposity measures for postoperative infection in elective spinal surgery BMI: body mass index; SSI: surgical site infection; CDC: Centers for Disease Control and Prevention; SAI: spine adipose index; PNI: prognostic nutritional index

Author	Year	Surgery type	BMI outcome	Adiposity outcome	Infection measure	Key conclusion
Donnally et al. [9]	2022	Posterior cervical fusion	Not predictive (p=0.153)	Subcutaneous fat and lamina-skin thickness (p<0.05)	SSI (CDC criteria)	Local adiposity metrics significantly predict SSI
Gupta et al. [10]	2021	Posterior lumbar fusion	Not predictive (p>0.05)	SAI ≥0.51 doubles risk (p=0.029)	Deep SSI	SAI strongly predictive; BMI lacks independent association
Lee et al. [11]	2016	Lumbar spine surgery	Not predictive in multivariate model	Fat thickness >50 mm=4× SSI risk (p=0.026)	SSI	Subcutaneous fat remains independent predictor over BMI
Mehta et al. [12]	2012	Posterolateral lumbar fusion	Obesity threshold predictive; BMI not	Subcutaneous fat and lamina-skin distance (p<0.05)	SSI	Regional fat metrics outperform BMI
Mehta et al. [13]	2013	Posterior cervical fusion	Not predictive (p=0.86)	Fat ratio >0.419=6× SSI risk (p=0.015)	SSI	Subcutaneous fat ratio strongly predictive; BMI not useful
Yang et al. [14]	2023	Posterior lumbar interbody fusion	Correlated but not predictive	Relative fat thickness and low PNI independently predictive	SSI	Dual risk from local adiposity and poor nutrition
Li et al. [15]	2019	Posterior lumbar fusion	Predictive in univariate, not multivariate	Subcutaneous fat thickness (p<0.001)	SSI	Fat thickness better predictor

In cervical spine procedures, Donnally et al. [9] found that lamina-skin thickness was significantly associated with SSI (p<0.05), while BMI lacked predictive utility (p=0.153). Mehta et al. [13] similarly showed that a subcutaneous fat ratio >0.419 conferred a sixfold increase in infection risk (p=0.015), whereas BMI had no statistical impact. For lumbar spine procedures, Gupta et al. [10] introduced the spine adipose index (SAI), with values ≥0.51 associated with doubled infection risk (OR=2.0), while BMI again failed to demonstrate an independent association. Mehta et al. [12] found that subcutaneous fat thickness at L4 and lamina-skin distance were predictive of SSI, but BMI was only significant when dichotomised by obesity thresholds.

Lee et al. [11] reported that L4 subcutaneous fat thickness above 50 mm led to approximately four times the infection risk (OR=4.0; p=0.026), solidifying fat depth as an independent marker. Yang et al. [14] expanded the perspective by incorporating the prognostic nutritional index (PNI), identifying both PNI and relative fat thickness as independent SSI predictors. Li et al. [15] reaffirmed the pattern: BMI was significant in univariate models but lost significance after adjustment, while subcutaneous fat thickness retained predictive value.

Details of each study's statistical design are compiled in Table 3, including surgical focus, adiposity metrics, ORs, infection definitions, and sample sizes. Most studies used CDC-based definitions for SSI, and several incorporated multivariate analyses to control for confounding factors such as nutritional status, comorbidities, and surgical duration. The table highlights the consistent trend: localised adiposity measures outperform BMI in predicting postoperative infection risk.

**Table 3:** Extracted study data for statistical analysis OR: odds ratio; CI: confidence interval; BMI: body mass index; SSI: surgical site infection; CDC: Centers for Disease Control and Prevention; SAI: spine adipose index; PNI: prognostic nutritional index

Study	Surgery type	Adiposity metric	Reported OR (with CI)	BMI predictive?	SSI definition	Sample size	Notes
Donnally et al. [9]	Posterior cervical fusion	Subcutaneous fat thickness	OR not explicitly given; p=0.026	Not significant (p=0.153)	CDC-defined SSI	205	Univariate significance only
Gupta et al. [10]	Posterior lumbar fusion	SAI ≥0.51	OR≈2.0 (not exact); p=0.029	Not significant	Deep SSI	42 (case-control)	SAI threshold defined
Lee et al. [11]	Lumbar spine surgery	Subcutaneous thickness at L4	OR=1.06; p=0.026	Not in multivariate model	CDC-defined SSI	149	Threshold-based
Mehta et al. [12]	Lumbar fusion	Subcutaneous thickness at L4	OR=1.037 per mm increase	Obesity predictive; BMI continuous not significant	SSI (CDC)	298	Adjusted analysis
Mehta et al. [13]	Cervical fusion	Fat ratio >0.419	OR=6.5; p=0.015	Not significant	CDC-defined SSI	213	Strong ratio cutoff
Yang et al. [14]	Posterior lumbar interbody	Relative fat thickness and PNI	OR=0.936 (fat ratio); OR=1.149 (PNI)	BMI not predictive	SSI (CDC)	766	Nutritional variables included
Li et al. [15]	Posterior lumbar fusion	Subcutaneous fat thickness	OR=1.383; p<0.001	BMI univariate only	SSI (CDC)	448	Multivariate model

Three studies [11,12,15] were identified as suitable for subgroup meta-analysis due to shared anatomical focus (lumbar spine), similar adiposity metrics, and common outcome definitions. Their data are presented in Table 4, which shows individual ORs and relative weights within the pooled analysis. Mehta et al. [12] reported an OR of 1.04 per millimetre increase in subcutaneous fat thickness; Lee et al. [11] reported an OR of approximately 1.06; and Li et al. [15] found an OR of 1.38 in a multivariate model.

**Table 4:** Summary of OR for subcutaneous fat thickness predicting SSI in lumbar spine surgery OR: odds ratio; CI: confidence interval; SSI: surgical site infection

Study	OR (95% CI)	Weight (%)
Lee et al. [11]	1.06 (1.02-1.10)	~42%
Mehta et al. [12]	1.037 (1.007-1.068)	~44%
Li et al. [15]	1.383 (1.178-1.623)	~14%

**Figure 3:**
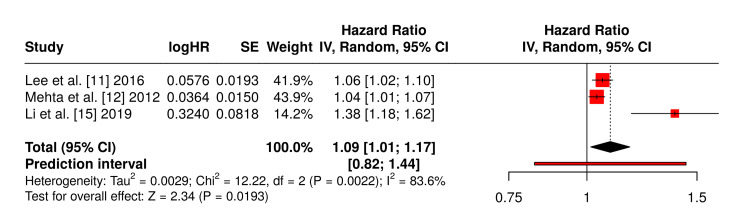
Pooled effect of subcutaneous fat thickness on SSI risk SSI: surgical site infection

A forest plot (Figure 3) was generated using a DerSimonian-Laird random-effects model [16] to account for methodological differences and threshold heterogeneity; the pooled OR was 1.09 (95% CI: 1.01-1.17; p=0.0193), indicating a statistically significant increase in SSI risk among patients with higher localised fat burden. Significant heterogeneity (I²=84%) was observed, primarily attributable to differences in measurement protocols and statistical adjustment methods, but it affirms a consistent directional effect.

Overall, these results support the hypothesis that site-specific adiposity metrics, particularly subcutaneous fat thickness, offer stronger and more clinically relevant predictive power for postoperative infection than BMI. Their integration into preoperative assessments may enhance surgical planning and improve infection prevention strategies in spinal procedures.

Discussion

Rethinking BMI in Surgical Risk Stratification

The findings of this review challenge long-standing assumptions about BMI's role in surgical risk stratification, presenting a compelling case for anatomically precise adiposity metrics as superior predictors of postoperative infection. While BMI is entrenched in clinical workflows due to its simplicity, its conceptual flaws and anatomical blind spots make it ill-suited for procedures like spine surgery, where operative field depth, regional fat deposition, and wound healing environments play critical roles in complication risk. Rothman [4] underscores BMI's insensitivity to body composition and fat distribution, noting that it conflates lean mass with adipose tissue and lacks granularity where it matters most.

Anatomical Precision and Predictive Value

Across the reviewed studies, site-specific adiposity measures consistently outperformed BMI in predicting SSI. What sets these metrics apart is their direct relevance to the anatomical zone of surgical intervention. Subcutaneous fat thickness, lamina-to-skin distance, and adiposity ratios offer tangible, reproducible data about the tissue envelope encountered during spine surgery. In contrast, BMI provides an abstract, body-wide index that fails to reflect localised biomechanical and inflammatory environments [18]. This anatomical proximity not only enhances predictive accuracy but also equips surgeons with actionable insights during preoperative planning.

Thresholds and Emerging Metrics

Threshold-based markers such as a subcutaneous fat thickness greater than 50 mm [11] or a fat-to-lamina ratio above 0.419 [13] present meaningful cutoffs for infection risk. These thresholds can be incorporated into surgical decision-making tools and electronic medical record systems to triage wound closure strategies and anticipate drain durations. Gupta et al. [10] advanced this paradigm by introducing the SAI, a reproducible and scalable metric that normalised fat thickness across spinal levels. Building on this framework, Shen et al. [19] proposed the subcutaneous lumbar spine index (SLSI), integrating subcutaneous fat thickness and spinous process height. An SLSI ≥0.7 was associated with a fourfold increase in early SSI risk (OR=4.23; p=0.012) and offered superior predictive performance over subcutaneous fat thickness alone (AUC 0.753 vs. 0.708). These findings reinforce the move toward anatomically nuanced, radiologically derived metrics in SSI risk stratification.

Meta-Analytic Evidence for Regional Adiposity

Beyond isolated findings, this review's focused meta-analysis of subcutaneous fat thickness in lumbar spine procedures [11,12,15] substantiates the independent association between regional adiposity and SSI risk. The pooled OR of 1.09 (95% CI: 1.01-1.17; p=0.0193) confirms a statistically significant increased likelihood of infection among patients with elevated fat thickness. Importantly, this finding withstands methodological variability and supports prior clinical observations that excess local fat impairs wound healing by reducing tissue perfusion and increasing dead space [20].

Inclusivity and Applicability in Complex Populations

Importantly, anatomically targeted adiposity metrics also offer greater inclusivity, particularly in atypical or underserved patient populations. Sukkarieh et al. [5] demonstrated how radiographic fat ratios provided a viable alternative to BMI in non-ambulatory paediatric patients with neuromuscular scoliosis, in whom traditional height and weight measurements are often impractical or inaccurate. Localised radiographic markers thus extend predictive power to complex cases traditionally excluded from standard risk models [21].

Nutritional Status as a Synergistic Risk Factor

In addition to fat distribution, nutritional status surfaced as a synergistic predictor of infection risk. Yang et al. [14] showed that poor nutrition, reflected by low PNI scores, compounded the SSI risk when paired with elevated local adiposity. These insights affirm that vulnerability is multifactorial and that predictive models should incorporate layered variables, including albumin levels, diabetes status, and duration of drainage, to construct more comprehensive risk assessments [22]. 

Integrating Metrics into Surgical Planning

Moreover, incorporating radiographic adiposity metrics into composite infection risk models enables tailored intraoperative and postoperative strategies. For example, high-risk patients identified preoperatively could benefit from prophylactic negative-pressure wound therapy. Shaffrey et al. [23] explored this approach using CT-derived morphometric data to guide preoperative counselling and surgical preparedness, advocating for the integration of imaging markers with standard lab results and patient history.

Need for Standardisation and Measurement Consistency

To fully realise the potential of these metrics, standardisation remains essential. Across the reviewed literature, measurement techniques varied. Some studies relied on CT or MRI-based thickness assessments, while others used manual callipers or intraoperative estimates. Such heterogeneity hinders cross-study comparisons and dilutes clinical confidence in these promising tools.

Toward Individualised, Anatomy-Informed Risk Models

Ultimately, this review signals a necessary evolution in surgical risk assessment, one that moves away from generic, population-level indicators like BMI toward individualised, anatomy-informed models. The adoption of local adiposity metrics, reinforced by reliable imaging techniques and supported by robust statistical validation, offers a path to more precise, equitable, and clinically effective care in spinal surgery. When paired with nutritional and comorbidity profiling, these tools promise to reshape infection prevention strategies and empower proactive surgical planning across diverse patient populations.

## Conclusions

This systematic review underscores the inadequacy of BMI in accurately predicting postoperative infection following elective spinal surgeries. In contrast, site-specific adiposity measures, particularly subcutaneous fat thickness and fat-to-skin depth ratios, demonstrated consistent and independent associations with SSI across both adult and paediatric populations. A subgroup meta-analysis of three studies yielded a pooled OR of 1.09, confirming the strength of this association and reinforcing subcutaneous fat thickness as a statistically significant and clinically meaningful predictor of infection risk.

These findings support a re-evaluation of preoperative risk assessment practices, advocating for the integration of anatomically localised adiposity metrics in place of or alongside BMI. Such a shift could improve patient stratification, inform surgical planning, and guide targeted infection prophylaxis strategies. Future studies should aim to standardise adiposity measurement protocols, validate threshold values across spinal procedures, and explore synergistic risk factors such as nutrition and systemic disease burden. Above all, the field must transition toward individualised, anatomy-informed risk models that reflect the complex realities of surgical care.
